# Therapeutic potential and research progress of microecological agents in kidney diseases

**DOI:** 10.3389/fmed.2026.1815214

**Published:** 2026-07-29

**Authors:** Yixiang Qian, Chenli Zhang

**Affiliations:** 1 Department of Nephrology, The Affiliated Hospital of Southwest Medical University, Luzhou, China; 2 Department of Nephrology, The Second People's Hospital, Yibin, China

**Keywords:** chronic kidney disease, gut microbiota, gut-kidney axis, kidney diseases, microecological agents

## Abstract

Patients with kidney disease often exhibit pathological alterations such as gut dysbiosis, accumulation of uremic toxins, and systemic inflammation. In recent years, microecological agents—represented by pre-biotics, probiotics, and synbiotics—have emerged as a novel strategy targeting the “gut-kidney axis.” These agents act through mechanisms including modulation of gut microbiota balance, improvement of intestinal barrier function, reduction of endotoxin levels, and anti-inflammatory as well as antioxidant effects. Our review of the available evidence on microecological agents across various kidney diseases reveals that these interventions can delay the progression of renal function decline in patients with Chronic Kidney Disease (CKD) by restoring gut microbiota balance and reducing the systemic burden of uremic toxins and other harmful metabolites. Concurrently, they help mitigate CKD-related complications, including mineral and bone disorders, cardiovascular and cerebrovascular events, and hyperuricemia. Moreover, microecological agents have demonstrated beneficial effects in attenuating renal injury in acute kidney injury (AKI), reducing the incidence of nephrolithiasis, and exerting a modulatory role in lupus nephritis (LN). However, existing evidence remains unevenly distributed, and conclusions regarding efficacy vary due to differences in the type, dosage, and timing of microecological interventions, thus necessitating a systematic review. This article synthesizes current research on microecological agents across various kidney diseases, systematically evaluates their clinical efficacy and heterogeneity, and discusses future development strategies and translational potential.

## 1 Introduction

Kidney diseases pose a major challenge to global public health systems. The spectrum of renal disorders is broad, ranging from common Chronic Kidney Disease (CKD) to acute and critical conditions such as acute Kidney Injury (AKI). All of these impose a heavy burden on human health and socioeconomic development. Among them, CKD has attracted particular attention due to its high prevalence (affecting approximately 9% of the global population) and significant associated disability and mortality (1). CKD is not only an important risk factor for cardiovascular events but also a leading cause of end-stage renal disease, ultimately requiring costly renal replacement therapy to sustain life. Concurrently, the incidence of AKI among hospitalized patients can be as high as 20%. AKI itself carries a high mortality risk and is a critical event that accelerates the progression of CKD and worsens long-term prognosis (2). A common feature underlying these diseases is that their onset and progression are closely intertwined with pathophysiological processes such as inflammation, oxidative stress, and disruption of internal homeostasis.

**Figure 1:**
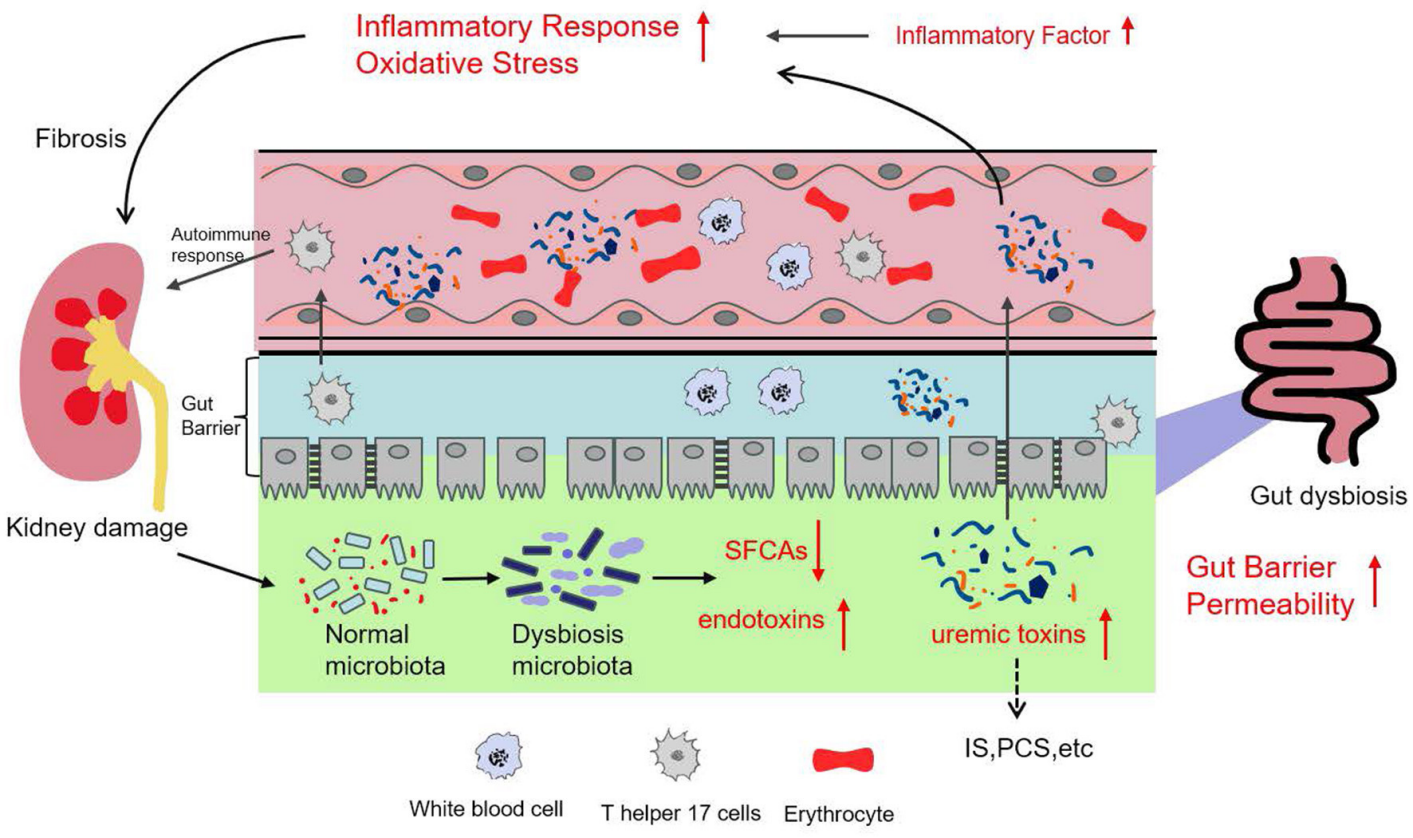
Gut-kidney axis.

Traditional perspectives in nephrology research have largely focused on pathological changes within the kidneys themselves. With the rapid advancement of microbiome science, it has become increasingly recognized that the human body functions as a complex ecosystem, in which interactions between distant organs play crucial roles in disease progression. Among these, the “gut-kidney axis” has emerged as a major focus in kidney disease research, with its pathophysiological significance becoming increasingly prominent (2, 3). The establishment of this theory is grounded in the following key evidence (Figure 1): first, patients with kidney disease exhibit gut dysbiosis, characterized by a reduction in beneficial bacteria and their metabolites—such as short-chain fatty acids, an increase in opportunistic pathogens, and an overall decline in microbial diversity. Second, this dysbiotic gut microbiota compromises the integrity of the intestinal mucosal barrier, facilitating the translocation of endotoxins (e.g., lipopolysaccharide) and gut-derived uremic toxins (e.g., indoxyl sulfate, p-cresyl sulfate) into the systemic circulation (3). Third, these gut-derived toxins exacerbate renal inflammation and oxidative stress, directly injuring kidney cells, and also activate systemic immune responses, thereby creating a vicious cycle of “gut toxin accumulation—systemic microinflammation—sustained deterioration of renal function.”

This understanding shifts the therapeutic target from the kidney itself to the intestinal microenvironment. Building on this foundation, the administration of microecological agents—such as pre-biotics, probiotics, and synbiotics—to restore a healthy gut microbiota, repair the intestinal barrier, and reduce the production of harmful substances, thereby indirectly protecting the kidneys, has emerged as a highly promising novel therapeutic approach. Although decisive evidence in this field is yet to be established, numerous pre-clinical and clinical studies have explored the application of microecological agents in various types of kidney diseases. Their conclusions exhibit a certain degree of heterogeneity, and the specific mechanisms of action, optimal application strategies, and long-term efficacy remain to be fully summarized and elucidated.

This review aims to systematically summarize the research progress on the association between microecological agents and kidney diseases. The article will first elaborate on the physiological functions of the gut microbiota and its role in kidney injury following dysbiosis. It will then discuss the specific evidence for the application of microecological agents in non-dialysis CKD, dialysis-dependent CKD, CKD-related complications, and other renal conditions (such as AKI, kidney stones, and lupus nephritis), while providing an objective analysis of current controversies and limitations in clinical practice. Finally, we will outline future directions for the development of microecological agents, with the goal of providing a clear reference framework and novel insights for both research and clinical practice in this field.

## 2 Physiological functions of the gut microbiota

The gut microbiota of healthy individuals is pre-dominantly composed of beneficial phyla such as Firmicutes and Bacteroidetes (4). These beneficial bacteria participate in various essential physiological and metabolic processes in the human body. Examples include the synthesis of Vitamin K and B-complex vitamins, the breakdown of plant cellulose, the activation of certain bioactive food components (e.g., flavonoids, isoflavones, and plant lignans), the degradation of dietary oxalate, and the biotransformation of conjugated bile acids (5, 6). The gut microbiota also reinforces intestinal barrier function and provides resistance against pathogenic infection through multiple mechanisms. These include repairing the structure of tight junction proteins, upregulating mucin gene expression, stimulating epithelial heat shock proteins, and competing with pathogens for binding sites on intestinal epithelial cells (7–9). Furthermore, the gut microbiota helps suppress intestinal inflammation and stress-induced injury via pathways mediated by Toll-like receptors (TLRs) (10, 11), which also contributes to maintaining the integrity of the intestinal epithelial barrier and epithelial homeostasis.

Short-chain fatty acids (SCFAs, such as acetate, propionate, and butyrate), produced by gut microbiota metabolism, are crucial for maintaining host health. Within the intestinal lumen, they reduce protein fermentation, protect the mucosa, and enhance barrier function by lowering luminal pH and nourishing colonic epithelial cells. Following their absorption from the colon into the systemic circulation, SCFAs exert broad, systemic regulatory effects. These include modulating immune and inflammatory responses and influencing energy metabolism and blood pressure. These systemic effects, particularly their potent anti-inflammatory and immunomodulatory capabilities, ultimately converge on the kidneys. They can alleviate systemic and local renal microinflammation and mediate renoprotective effects via specific receptors. Consequently, SCFAs have demonstrated the potential to prevent AKI and delay the progression of CKD in animal models (12–16).

## 3 Gut dysbiosis and renal injury

When gut dysbiosis occurs, the normal physiological functions of the microbiota are consequently diminished, thereby impairing key host processes such as nutrient metabolism and immune regulation. This functional disturbance disrupts the body's internal homeostasis and can ultimately lead to damage across multiple organ systems.

Following dysbiosis, the production of uremic toxins in the gut increases. This contributes to the disruption of the intestinal epithelial barrier and a reduction in renal clearance, leading directly to renal injury (3, 17, 18). Furthermore, it imposes a persistent burden of inflammation, oxidative stress, and cardiovascular complications on patients with kidney disease. Concurrently, the decrease in SCFA production and the compromised intestinal mucosal barrier allow more gut-derived uremic toxins to enter the systemic circulation, which in turn exacerbates kidney damage. Additionally, gut dysbiosis and its associated disease metabolites can activate immune responses. For instance, gut-derived Th17 cells can migrate to the kidneys, triggering autoimmune reactions that influence the course of renal disease (19).

In patients with CKD, alterations in the gut microbiota occur, characterized by a marked decrease in both the abundance and diversity of fecal microbiota (20). These changes, in turn, exacerbate renal fibrosis and oxidative stress (21).

## 4 Concept of microecological agents and their role in the gut-kidney axis

Microecologics refer to a category of preparations that promote health by modulating the host's intestinal microecosystem. This category encompasses probiotics, pre-biotics, synbiotics, post-biotics, and others. These agents can exert renoprotective effects primarily through mechanisms such as improving the balance of the gut microbiota, inhibiting the growth of pathogenic bacteria, and modulating immune responses (22, 23).

Pre-biotics are non-digestible dietary components that function by selectively stimulating the proliferation of beneficial intestinal bacteria, such as Bifidobacterium and Lactobacillus, thereby modulating host metabolism and immune responses (24). They represent an emerging tool for improving gut-kidney axis function. Clinically commonly used pre-biotics include fructooligosaccharides (FOS), inulin, resistant starch (RS), and lactulose. Their mechanisms of action encompass reducing uremic toxin production, restoring the intestinal barrier, suppressing systemic inflammation, and modulating oxidative stress (17, 25–27).

Probiotics are consortia of live microorganisms that, when administered in adequate amounts through dietary intake, confer a health benefit on the host by improving intestinal health. Common probiotic preparations include strains from genera such as Bifidobacterium and Lactobacillus. Their beneficial actions involve modulating the composition of the gut microbiota, attenuating inflammatory responses, enhancing the integrity of intestinal tight junctions, and reducing the production of gut-derived uremic toxins (28).

Synbiotics refer to combinations of pre-biotics and probiotics. As an emerging class of combined microecological agents, they have demonstrated effects superior to those of individual probiotics or pre-biotics alone (25, 29, 30). Furthermore, the administration of synbiotics has been shown to reduce levels of insulin resistance markers and the prevalence of metabolic syndrome in elderly patients. This modulation may consequently lower the incidence of diabetic complications, including diabetic nephropathy (31).

Post-biotics are preparations composed of inanimate microorganisms and their components that confer health benefits on the host. They offer superior stability compared to probiotics (32). The mechanisms of action of post-biotics include modulating the gut microbiome and its metabolic derivatives, enhancing the intestinal epithelial barrier, and regulating both immune and neural responses (32). Currently, numerous clinical trials involving post-biotics have been conducted across various conditions, including irritable bowel syndrome, diarrhea, cancer, respiratory diseases, and tuberculosis. However, the supporting evidence remains limited, and in particular, few human studies have specifically investigated the relationship between genuine post-biotics and CKD (33).

## 5 Microecological agents and kidney diseases

### 5.1 Microecological agents and non-dialysis chronic kidney disease

In delaying the progression of CKD, microecological agents demonstrate multifaceted potential. The core mechanism lies in modulating the gut microbiota to reduce the accumulation of uremic toxins and alleviate systemic microinflammation and oxidative stress, thereby ameliorating renal function and pathological injury.

The strain Lactobacillus reuteri GMNL-263 was observed to inhibit hyperglycemia-induced renal fibrosis in diabetic rats (34). In diabetic nephropathy patients, an 8-week intake of soymilk enriched with Lactobacillus A7 significantly reduced levels of cystatin C and the inflammatory marker progranulin, thereby alleviating the inflammatory state and improving renal function (34). In an adenine-induced CKD rat model, lactulose was shown to reduce the area of renal fibrosis, modulate the gut microbiota, and lower levels of uremic toxins. Concurrently, it decreased serum markers of oxidative stress, such as advanced oxidation protein products and malondialdehyde, while elevating antioxidant substances like glutathione. These combined anti-inflammatory and antioxidant effects contribute to delaying the progression of renal impairment (35). Synbiotics and dietary fiber can modulate the gut microbiota, leading to an increased relative proportion of SCFAs (36). This, in turn, reduces the production of uremic toxins, improves intestinal barrier function, and is associated with a lower prevalence of chronic kidney disease (37). Supplementation with exogenous SCFAs has also been proven to directly improve renal function (16). The combination of inulin with a low-salt, low-protein diet has been shown to effectively reduce serum levels of uremic toxins in CKD patients, decrease the source of intestinal inflammation, and alleviate systemic inflammatory responses (38). RS, by increasing the abundance of the genus Faecalibacterium in feces, lowers the levels of inflammatory factors and blood urea nitrogen concentration (39). It also reduces plasma thiobarbituric acid reactive substances and pro-inflammatory cytokine levels, thereby exerting anti-inflammatory effects and delaying the progression of renal impairment (40, 41). By the way, RS combined with exercise training may exert a synergistic anti-inflammatory effect (41). In addition to reducing serum interleukin-6 (IL-6) levels in CKD patients, FOS also decrease systemic levels of uremic toxins and mitigate inflammatory responses (26, 42). While improving the gut microbiota, synbiotics have been shown to lower high-sensitivity C-reactive protein (hs-CRP) levels and increase estimated glomerular filtration rate (eGFR), without demonstrating significant effects on serum potassium, blood urea nitrogen, IL-6 levels, or proteinuria (43). ß-Glucan can reduce gut-derived uremic toxins and positively influence the microbiota, thereby contributing to a certain degree of renal function improvement (44). Furthermore, observational studies suggest that the intake of probiotic, pre-biotic, or yogurt supplements is associated with a reduced risk of CKD progression, an effect that may be more pronounced in women and individuals aged 55 years or older (1).

However, some studies have also reported negative findings. For instance, certain research indicates that microecological agents do not exhibit a significant effect on lowering serum creatinine levels (45). Additionally, arabinoxylan oligosaccharides have shown no impact on uremic retention solutes or insulin resistance in CKD patients (46). These conflicting conclusions suggest that the efficacy of microecological agents may be influenced by multiple factors, including the type of preparation, study design, and baseline characteristics of the patients involved.

### 5.2 Microecological agents and dialysis-dependent end-stage renal disease

Intervention with microecological agents has been shown to significantly reduce serum levels of p-cresyl sulfate (PCS), endotoxin, malondialdehyde (MDA), and inflammatory markers in hemodialysis (HD) patients, while increasing total antioxidant capacity and glutathione levels (47). The efficacy of different types of microecological agents varies slightly in dialysis patients. Pre-biotics show advantages in reducing IL-6, blood urea nitrogen (BUN), tumor necrosis factor-alpha (TNF-α), and uremic toxins. Probiotics demonstrate superior efficacy in alleviating gastrointestinal symptoms, whereas synbiotics are particularly effective in lowering CRP and endotoxin levels (29). The use of RS helps decrease serum levels of pro-inflammatory cytokines in HD patients, contributing to an anti-inflammatory effect (48). Intercellular adhesion molecule-1 (ICAM-1) and vascular cell adhesion molecule-1 (VCAM-1) are key mediators of endothelial dysfunction in CKD patients. Multiple studies have confirmed elevated serum concentrations of ICAM-1 and VCAM-1 in HD patients (49–51). Synbiotics can reduce serum levels of ICAM-1 and VCAM-1 in these patients, which is beneficial for mitigating vascular endothelial dysfunction (52). Inulin enriched with FOS can significantly lower serum PCS levels—an independent predictor of cardiovascular disease in hemodialysis (HD) patients (53). However, whether this reduction translates into improved cardiovascular outcomes requires further confirmation. Furthermore, a symbiotic gel formulated with pre-biotics and probiotics has been reported to improve common gastrointestinal symptoms (GIS) in dialysis patients and demonstrates a reasonable safety profile (54). In hemodialysis patients, the application of synbiotics yields greater benefits compared to probiotics or pre-biotics alone. For instance, synbiotics are superior to probiotic supplementation alone in improving endothelial function and reducing the occurrence of related cardiovascular risk events in this population (25). Their effect on ameliorating anxiety and depressive symptoms in HD patients is also more pronounced (30). A single-center, non-randomized, open-label phase I/II clinical study (17) conducted in maintenance hemodialysis patients showed that inulin enriched with FOS resulted in a significant 20% reduction in the median serum concentration of PCS and an approximately 20% decrease in the mean production rate of PCS. However, no significant changes were observed in the production rate or serum concentration of indoxyl sulfate (ICS). Additionally, synbiotics can reduce serum levels of hs-CRP, anti-heat shock protein 70 antibodies, and endotoxin in HD patients, thereby decreasing the incidence of complications such as infections (25).

Intervention with inulin-type fructans (ITFs) can limit the production of microbial indole in the gut of peritoneal dialysis (PD) patients, lower intestinal pH, and reduce the generation of uremic toxins, thereby contributing to a deceleration of renal function decline to some extent (55). Furthermore, a daily dosage of 10 g/d of ITFs significantly reduces the circulatory retention of arsenic (As) in PD patients, increases urinary and dialytic clearance of As, elevates the Firmicutes/Bacteroidetes ratio (which is positively correlated with fecal uric acid degradation), raises fecal levels of SCFAs (such as butyrate), and enhances the excretion of primary bile acids (56). However, this regimen is insufficient to lower plasma trimethylamine N-oxide (TMAO) levels in these patients (57).

### 5.3 Microecological agents and chronic kidney disease-related complications

Patients with CKD frequently develop hyperphosphatemia due to increased intestinal phosphate absorption, elevated bone turnover, and reduced renal phosphate excretion (58). Hyperphosphatemia not only adversely affects the diversity of the gut microbiota but also contributes to cardiovascular calcification, thereby increasing all-cause and cardiovascular mortality in CKD (59–61). In animal models of CKD, supplementation with synbiotics has been shown to ameliorate the composition of the gut microbiota, enhance the expression of tight junction proteins, improve intestinal barrier permeability, and help control serum phosphate and parathyroid hormone (PTH) levels (62). These effects collectively lead to an alleviation of vascular calcification and a reduction in the incidence of hyperphosphatemia and secondary hyperparathyroidism. Additionally, such supplementation lowers the risk of bacterial translocation from the gut into the systemic circulation. The use of specially formulated pre-biotics designed to increase their retention time and absorption capacity in the gut can significantly modulate the gut microbiota, reduce intestinal permeability, lower serum phosphate concentrations, increase fecal phosphate excretion, decrease urinary phosphate levels, and reduce serum levels of fibroblast growth factor-23 (FGF-23) and PTH (63). This intervention markedly improves vascular calcification and also positively influences lipid metabolism in rat models. Furthermore, although inulin does not alter renal function in CKD rats, it reduces serum phosphate and PTH levels, improves bone and cardiovascular parameters, lowers concentrations of gut-derived uremic toxins, modifies cecal microbiota composition, and slows the progression of chronic kidney disease-mineral and bone disorder (CKD-MBD) (64).

Chronic kidney disease is frequently complicated by cardiovascular and cerebrovascular disorders, including heart failure (HF), atherosclerosis, and stroke. In animal models, oral supplementation with Bacteroides has been shown to reduce LPS production, successfully alleviate endotoxemia, suppress pro-inflammatory immune responses, and attenuate atherosclerotic plaque inflammation as well as its progression (65).

By measuring flow-mediated dilation in CKD patients, it was demonstrated that the application of FOS can protect endothelial function, with a more pronounced protective effect observed in patients with less severe endothelial injury (42). Furthermore, daily supplementation with Lactobacillus has been shown to improve endothelium-dependent vasodilation in arteries by enhancing nitric oxide bioavailability, while also reducing systemic inflammatory responses (66). Currently, a pre-clinical and clinical trial is underway that aims to improve heart failure associated with CKD by administering probiotics to reduce *Escherichia coli* abundance and IS levels, thereby ameliorating cardiac outcomes in rats and patients with chronic kidney disease (67).

A study conducted in a mouse model of ischemic stroke demonstrated that probiotic treatment reduced ischemic brain injury by 52% and improved neurological outcomes. The neuroprotective effects observed are likely attributable to the anti-inflammatory properties of probiotics and their ability to modulate oxidative stress-induced damage (68). Collectively, these findings suggest that the application of microecological agents in patients with CKD may offer potential benefits in preventing cardiovascular and cerebrovascular events or improving clinical outcomes.

Patients with CKD frequently present with anemia. The condition is multifactorial in nature, with contributing factors including elevated levels of uremic toxins, insufficient erythropoietin (EPO) production, shortened red blood cell lifespan, chronic inflammation and reduced levels of SCFAs (69, 70). Elevated serum levels of IS in patients with CKD have been shown to inhibit erythropoiesis through multiple mechanisms. These include inducing apoptosis in the UT7/EPO cell line, downregulating the expression of erythropoiesis-related genes—such as GATA-1, erythropoietin receptor (EPO-R), and β-globin—and suppressing the EPO-EPO-R signaling pathway. These effects impair erythroid differentiation capacity, thereby promoting cellular senescence and apoptosis during the erythropoiesis process (71, 72).

In a randomized controlled trial, dietary fiber was found to elevate hemoglobin levels in HD patients, an effect that may be associated with increased butyrate production resulting from gut microbiota alterations (36). In another study, 12 weeks of oral sodium propionate supplementation in HD patients resulted in decreased inflammatory parameters, reduced oxidative stress, lower ferritin levels, and an elevated transferrin saturation index, altaong with declines in serum levels of IS and PCS (73). However, in another randomized controlled trial involving patients with CKD who received a pre-biotic (fructooligosaccharide at 12 g/day for 3 months), although IL-6 levels decreased, no significant changes were observed in hemoglobin levels or IS concentrations (42).

Uric acid is currently recognized as a cardiovascular risk factor, and suggestions have been made to consider hyperuricemia as a potential risk factor for CKD (52, 74). Inulin-type pre-biotics can increase the intestinal excretion of uric acid, thereby lowering serum uric acid levels in PD patients. Concurrently, during pre-biotic intervention, the Firmicutes/Bacteroidetes ratio increases, enriching purine-degrading species within the gut microbiota (27). This shift contributes to reduced production of uremic toxins and a decreased risk of complications such as cardiovascular events in patients. However, the more in-depth physiological mechanisms by which microecological agents lower uric acid require further research for confirmation.

Constipation, as one of the common complications in PD patients, often compromises their quality of life. Studies suggest that treatment with FOS is effective for constipation in patients undergoing continuous ambulatory peritoneal dialysis (CAPD) (75). However, due to limitations in sample size and study duration, its safety profile and long-term efficacy require further validation.

### 5.4 Microecological agents and other renal diseases

#### 5.4.1 Microecological agents and AKI

Microecological agents can alleviate renal inflammation and damage to renal tubular epithelial cells by elevating levels of SCFAs in both serum and kidney tissue. They have been shown to correct gut microbiota dysbiosis induced by bilateral renal ischemia-reperfusion in mice and mitigate the resulting acute kidney injury (15). Currently, there is a paucity of both clinical studies and animal experiments investigating the application of gut microbiota modulators, such as these agents, in AKI. Research into the relevant mechanisms is also lacking. Future efforts should prioritize exploring the potential of gut microbiota modulators in this field.

#### 5.4.2 Microecological agents and nephrolithiasis

The primary pathological feature of nephrolithiasis is the deposition of crystalline substances in the kidneys due to various causes. Gut-derived oxalate is also recognized as a significant contributor to the development of nephrolithiasis. Evidence suggests that certain probiotics, particularly oxalate-degrading bacteria such as Oxalobacter species, can degrade intestinal oxalate and reduce urinary oxalate excretion, thereby decreasing the incidence of kidney stone formation (76–80). In addition to Oxalobacter, other probiotic strains, including those from the genera Lactobacillus and Bifidobacterium, have demonstrated similar effects (81–83). Given that the diversity and abundance of the gut microbiota vary under different dietary patterns, the efficacy of intestinal probiotics in preventing nephrolithiasis is consequently influenced by dietary factors (83).

#### 5.4.3 Microecological agents and lupus nephritis (LN)

Systemic lupus erythematosus (SLE) is a multisystem autoimmune disorder, with approximately half of affected patients developing renal involvement. LN represents a major cause of mortality in SLE. Alterations in the gut microbiota have been implicated in various autoimmune diseases, and emerging evidence suggests that patients with LN harbor a distinct microbial composition within the gastrointestinal tract (84–86). Consequently, the role of the gut microbiota in LN has garnered increasing attention. In an animal study, administration of Lactobacillus was shown to reduce intestinal IL-6 production and increase circulating IL-10 levels. This intervention suppressed immune responses in both female and castrated male mice with LN by decreasing levels of immunoglobulin G2a, which is considered a major immune deposit in the kidneys of MRL/LPR mice. Regarding clinical trials, further research is anticipated to elucidate the therapeutic potential of microecological agents in LN (87).

## 6 Controversies and limitations in the application of microecological agents

### 6.1 Heterogeneity in therapeutic efficacy

Although pre-clinical studies have demonstrated that pre-biotics exert renoprotective effects in animal models, their clinical translation has been marked by considerable heterogeneity. For instance, low-dose inulin (10 g/day) has been shown to reduce serum levels of uremic toxins and modulate inflammatory status in patients with CKD (38). In contrast, high-dose FOS (12 g/day), despite decreasing total serum indoxyl sulfate levels, failed to improve renal function (26). Furthermore, while lactulose is commonly used clinically to manage constipation in patients with CKD, studies have indicated no significant benefit in the prevention of peritoneal dialysis-related peritonitis (88). Due to variations in the types, dosages, and durations of microecological interventions across studies, findings have been inconsistent, with some trials reporting beneficial effects and others yielding negative results. These conflicting findings suggest that the therapeutic efficacy of microecological agents may be jointly influenced by factors such as disease type, timing of intervention, and baseline characteristics of the gut microbiota. Additionally, the specific strain or type of agent used, dosage, sample size, patient inclusion and exclusion criteria, and underlying etiology of CKD may also contribute to discrepancies in study outcomes. Future research should incorporate larger sample sizes, precisely define the types and dosages of microecological agents, and conduct more in-depth investigations to comprehensively explore the potential benefits of various microbial preparations in slowing CKD progression and reducing complication risks.

### 6.2 Dosage heterogeneity

Studies have indicated that a daily dosage of pre-biotics exceeding 5 g is sufficient to influence gut microbiota diversity. However, a threshold dosage of 15–20 g/day may be required to achieve a reduction in uremic toxin concentrations (89). Currently, most studies investigating gut microbiota modulators lack standardized dosage protocols, which may in part account for the variability observed in research findings.

### 6.3 Uncertainty regarding safety

The use of probiotics and pre-biotics may lead to adverse effects such as abdominal distension, diarrhea, and gut microbiota dysbiosis (75, 90). Most trials have not investigated the long-term outcomes of microecological agents, and the safety profile of these preparations remains inconclusive (Table 1). Currently, a feasibility study investigating the use of inulin in this population is underway, aiming to clarify the safety of this specific microecological agent in patients undergoing maintenance hemodialysis (91).

**Table 1:** Adverse reactions of some microecological agents.

References	Year	Participants	Sample size	Intervention	Outcome
(38)	2023	CKD (G3-5)	*N* = 45	Inulin	One subject with aggravated constipation
(54)	2015	HD	*N* = 22	Symbiotic Gel	Alleviated gastrointestinal reactions such as abdominal distension and constipation
(88)	2021	PD	*N* = 100	Lactulose	Diarrhea more common than in the control group
(42)	2021	CKD (G3-5)	*N* = 46	FOS	One subject with abdominal discomfort
(46)	2016	CKD (G3-5)	*N* = 40	Arabinoxylan Oligosaccharides	One subject with nausea
(36)	2022	CKD (G5)	*N* = 162	DF mixture	Aggravated gastrointestinal reactions including abdominal distension, diarrhea, and constipation

HD, hemodialysis; PD, peritoneal dialysis; Symbiotic gel: a mix of probiotics (Lactobacillus acidophilus NCFM and Bifidobacterium lactis Bi-07) and Inulin and omega-3 fatty acids and vitamins; DF, dietary fiber, a mix of galactomannan, resistant dextrin, FOS and starch.

### 6.4 Lack of targeting specificity

The current mode of action of microecological agents remains relatively broad-spectrum, primarily exerting a holistic regulatory effect on the gut microbiota. Future research breakthroughs may lie in the development of precision targeting technologies. Potential approaches include designing protective shells via chemical synthesis to enable site-specific delivery of therapeutic molecules, developing microecological agents that can be specifically utilized by key bacterial species, or targeting specific metabolic enzymes or receptors to precisely regulate the most relevant microbial metabolic pathways involved in kidney disease (such as uremic toxin generation). These strategies hold promise for achieving precise intervention along the gut-kidney axis.

## 7 Therapeutic potential of microecological agents in kidney diseases

### 7.1 Precision intervention strategies

To achieve precise modulation of the gut microbiota, the selection of pre-biotic types can be tailored based on specific gut microbiota characteristics, such as enterotype classification. For instance, inulin-type fructans (ITFs) have been shown to increase the Firmicutes/Bacteroidetes ratio, while resistant starch can enhance the abundance of Faecalibacterium in feces (39, 56). Lactulose, through its fermentation product lactic acid, creates a favorable environment for the survival of Lactobacillus. Furthermore, synbiotics formulated through strategic combinations of different probiotic and pre-biotic types may yield unexpected therapeutic benefits.

### 7.2 Combination therapy (composite formulations)

The progression of CKD and its complications arises from the interplay of multiple pathological mechanisms. For instance, renal fibrosis is closely associated with various factors, including the accumulation of uremic toxins, a state of microinflammation, and disturbances in phosphate metabolism. Multiple studies have indicated that the application of synbiotics in patients with CKD yields superior effects compared to the use of single microecological agents alone (25, 29, 30). The future development of novel composite formulations (e.g., combining pre-biotics, specific probiotics, and active ingredients with well-established renoprotective properties) holds significant promise. The core advantage lies in achieving multi-target therapeutic effects through synergistic interactions among different components. This concept has received preliminary validation in animal experiments. For example, one study prepared a microgel by combining the anti-fibrotic agents emodin and asiatic acid with *Lactobacillus casei* (92). This composite formulation not only targeted damaged kidneys more effectively and ameliorated fibrosis but also simultaneously modulated the gut microbiota, demonstrating superior efficacy compared to any single component alone. These findings provide theoretical validation and a feasible approach for the future development of combination therapies capable of both precisely regulating the intestinal microecology and directly intervening in key pathological processes such as renal fibrosis and vascular calcification.

### 7.3 Long-term outcome studies

Currently, research on microecological preparations mainly focuses on their short-term efficacy in subjects, while longitudinal studies on long-term outcomes are extremely scarce (Table 1). However, the long-term prognosis of patients with kidney disease is equally important, and more systematic studies on the long-term effects of microecological preparations are urgently needed in the future.

## 8 Conclusion

In summary, microecological therapies targeting the “gut-kidney axis” hold broad prospects. However, the heterogeneity of current evidence limits their clinical translation. Future core developments in this field should focus on in-depth exploration of their molecular mechanisms and the subsequent development of precise, individualized, multi-target therapeutic strategies.
